# Evaluation of the Role of Faecal Calprotectin in the Management of Psoriatic Patients under Treatment with Biologic Drugs

**DOI:** 10.3390/biomedicines10112968

**Published:** 2022-11-18

**Authors:** Eugenia Veronica Di Brizzi, Annachiara Rocco, Graziella Babino, Dario Buononato, Giuseppe Argenziano, Anna Balato

**Affiliations:** Dermatology Unit, University of Campania Luigi Vanvitelli, 80131 Naples, Italy

**Keywords:** calprotectin, faecal calprotectin, psoriasis, biological therapy, inflammatory bowel disease

## Abstract

**Background:** Fecal calprotectin has emerged as a significant, validated, and non-invasive biomarker allowing for the evaluation of inflammatory bowel disease. Our study assessed the reliability of the use of faecal calprotectin as a valuable tool in the management of psoriatic patients on biological therapy. **Methods:** This was a single-centre prospective study including adult patients affected by moderate-to-severe psoriasis starting biological therapy. Faecal calprotectin levels were evaluated at baseline and at week 24 (W24) of treatment in all enrolled patients. **Results:** Overall, 129 patients were enrolled. The mean baseline faecal calprotectin levels were 74.7 μg/g and a significant reduction was detected at W24 of biological therapy (57.5 μg/g). An analysis of faecal CP values stratified by therapy type was performed. No significant reduction was assessed at W24 for any of the anti-IL17 drugs, whereas a significant reduction was detected for all IL23 inhibitors. **Conclusions:** Our study showed the potential use of faecal CP levels as a valuable tool for exploring intestinal inflammation in the management of psoriatic patients undergoing treatment with biologic drugs.

## 1. Introduction

Psoriasis is a chronic, systemic immune-mediated disease affecting 2–4% of the population worldwide [1,2]. Clinically, psoriasis is characterized by development of erythematous, indurated, scaly, pruritic, often painful skin plaques typically occurring in a symmetrical distribution involving the elbows, knees, trunk and scalp [3], causing significant impact on health, wellbeing, and quality of life [4,5,6].

Nowadays, psoriasis is considered a systemic inflammatory disease with comorbidities affecting many organ systems. Psoriasis is associated with a greater number of comorbidities, including, but not limited to, psoriatic arthritis (PsA), cardiovascular disease, diabetes mellitus, obesity, inflammatory bowel disease (IBD) and nonalcoholic fatty liver disease compared with the general population [7].

In particular, an increased prevalence as well as incidence of IBD among patients with psoriasis has been observed [8,9] and the prevalence of psoriasis in IBD patients was reported at 1.2% in a recent meta-analysis [10].

Psoriasis onset is triggered when genetic and/or environmental factors activate plasmacytoid dendritic cells, resulting in the production of numerous proinflammatory cytokines, including tumour necrosis factor (TNF)-α, interferon (IFN)-γ, interleukin (IL)-17, IL-22, IL-23 and IL-1β [11]. Many of these cytokines stimulate keratinocyte hyperproliferation, which perpetuates a cycle of chronic inflammation [12].

Mechanistically, psoriatic lesions result from hyperproliferation and disturbed differentiation of epidermal keratinocytes that are provoked by immune mediators of the IL-23/IL-17 pathway [7,13].

IL-23 is produced by dendritic cells and promotes Th17/Th22 cell proliferation and activation, which in turn produces IL-17 and IL-22. This represents a central pathway in the pathogenesis of psoriasis, as evidenced by the efficacy of biologic treatments targeting this pathway in psoriasis. In contrast, inhibition of IFN-γ, the main cytokine produced by Th1 cells, showed only moderate effects in psoriasis, despite the strong IFN-γ signature in psoriasis skin lesions [14,15].

The development of biological agents, such as anti-TNFα, anti-IL17 and anti-IL23 antibodies offers a potentially safer and long-term option for patients with moderate-to-severe psoriasis [16,17].

A strong relationship is known to exist between the skin and gastrointestinal inflammation, as evidenced by a higher prevalence of psoriasis and inflammatory bowel disease in the same subjects. Moreover, it has been reported that patients with PsA and patients with skin psoriasis show a lower relative abundance of multiple intestinal bacteria compared to healthy controls. Although some genera were concomitantly decreased in both conditions, PsA subjects had a lower abundance of reportedly beneficial taxa. This particular gut microbiota profile in PsA was similar to that previously described in patients with inflammatory bowel disease [18]. It is now established that alterations in the intestinal microbiome can cause an increase in intestinal permeability with a consequent increase in intestinal inflammation [18,19].

Calprotectin (CP) has recently been considered a significant endogenous danger signal or Damage-associated molecular patterns (DAMPs) and an excellent biomarker for inflammatory processes that can trigger intracellular signalling cascades in adjacent cells, leading to the release of essential mediators of inflammation and initiation of inflammatory processes [20,21].

CP is an abundant cytosolic protein complex (comprising S100A8 and S100A9), representing ~45% of total cytosolic protein [22]. CP is capable of binding calcium and zinc, which are ubiquitous minerals in the human body, but is mainly present within neutrophil granulocytes, monocytes and macrophages. While carrying out their defensive functions, these cells can trigger an inflammatory response towards dangerous foreign agents. The normal function of CP is therefore to counteract the development of bacteria and fungi within the body [23,24].

It has been widely reported that CP levels in body fluids tend to increase in correspondence with inflammatory phenomena [23,25]. Therefore, CP can act as an indirect marker of inflammation.

In particular, in IBD, the concentration of faecal CP rises significantly compared to the norm and is about six-times greater in concentration than that of plasma and represents an extremely sensitive parameter for the diagnosis of IBD [24,26]. Therefore, this protein is recognized as a suitable biomarker for monitoring the response to therapy [27,28,29].

Our study evaluated the reliability and clinical value of faecal CP as a tool for use in the management of psoriatic patients on biological therapy.

## 2. Materials and Methods

This was a single-centre prospective study including adult patients affected by moderate-to-severe psoriasis starting biological therapy. Inclusion criteria were patients of both sexes, age ≥ 18 years, with diagnosis of psoriasis (Psoriasis Area Severity Index-PASI ≥ 10) starting any biological therapies. The study population included biologic-naïve subjects and patients who failed to respond adequately to previous interventions. All patients were treated in monotherapy with anti-TNFα, anti-IL17, or anti-IL23 drugs at the approved dosage for plaque psoriasis, after appropriate screening tests. The approved dose regimen for plaque psoriasis was also administered to patients affected by concomitant PsA and or IBD. Exclusion criteria were active tuberculosis (TB), hepatitis B surface Ag positive, hepatitis C ab positive and malignancy except treated basal cell carcinoma, and presence of other skin conditions that would interfere with evaluations. All the following data were collected: demographic data (e.g., age, sex, height, weight and body mass index [BMI]), disease duration, previous systemic therapies, associated co-morbidities (such as PsA, diagnosed according to CASPAR [Classification criteria for Psoriatic Arthritis] and IBD. PASI score and faecal CP levels were evaluated at baseline and after week 24 (W24) of treatment in all enrolled patients.

### 2.1. Faecal Calprotectin

Stool samples for assessment of faecal calprotectin levels were obtained from all study participants at baseline, and after 24 weeks of treatment. Samples were frozen at −70 °C and batch analysed via ELISA. The test used was CalproLab ELISA (ALP) CALP0170 (Svar, Lysaker, Norway). This product uses ALP for signalling and is thus measured at 405 nm. The range of the test is 25–2500 mg/kg.

### 2.2. Statistical Analysis

Data were analysed and Wilcoxon matched pairs test or Mann–Whitney test was used to calculate statistical differences. Correlations were evaluated with non-parametric Spearman’s rho test. Statistical analyses were performed using GraphPad Prism 6.0 (GraphPad Software Inc, La Jolla, CA, USA). Values of *p* < 0.05 were considered statistically significant.

## 3. Results

### 3.1. Demographic and Clinical Characteristics of Patients

Overall, 129 patients were enrolled. Most were male (57%, 73/129) with a mean age of 54 ± 13.6 and mean BMI of 28.3 ± 5.8. Co-morbid conditions were reported in 32% (41/129) of patients. It is worth noting that 16.15% (21/129) were affected by PsA and 5.28% (7/129) by IBD. PASI score at baseline was 17 ± 4.8.

Only 32% of patients (41/129) were bio-naïve. The three main biological class drugs were started and distributed as follows: 64% (83/129) on anti-IL17 (ixekizumab 30/129, secukinumab 45/129 and brodalumab 8/129), 30% (39/129) on anti-IL23 (guselkumab 19/129, tildrakizumab 8/129 and risankizumab 12/129) and 2% (3/129) on anti TNF-α (adalimumab 3/129) (Table 1).

### 3.2. Faecal CP Level Analysis

**Figure 1:**
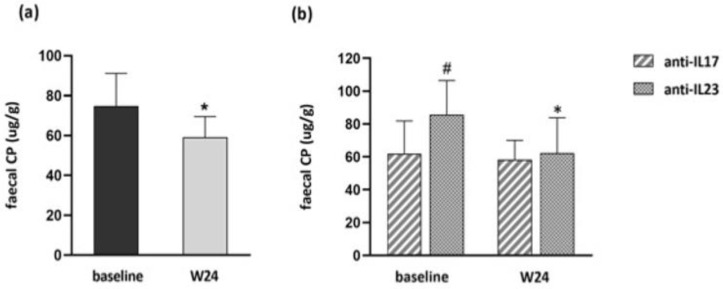
(**a**) Mean baseline and W24 faecal CP levels. (**b**) Mean baseline and W24 faecal CP levels stratified by anti-IL17 and anti-IL23 treatments. (**a**,**b**) Data are displayed as mean ± SD. Statistical analysis was performed with Wilcoxon matched pairs test: * *p* < 0.05 with respect to baseline; Mann–Whitney test: # *p* < 0.05 with respect to subjects on anti-IL17 drugs.

The most common cut-off value for faecal CP is 50 µg/g for a negative test. The mean baseline faecal CP levels were 74.7 μg/g and a significant reduction (*p* < 0.05) was detected at W24 of biological therapy (57.5 μg/g) (Figure 1a). Mean baseline levels in patients starting treatment with anti-IL17 and anti-IL23 drugs were 61.8 μg/g and 85.7 μg/g, whereas at W24 they were 56 μg/g and 62.3 μg/g, respectively. Significantly higher baseline values in subjects starting anti-IL23 with respect to those starting anti-IL17 were detected. However, a significant reduction was assessed at W24 only in subjects on anti-IL23 (*p* < 0.05) (Figure 1b).

A significant PASI score reduction was detected at W24 (*p* < 0.01) with a mean score of 4.5 ± 2.2. No correlation between disease severity and faecal CP levels were found either before or after W24 of treatment (data not shown).

**Figure 2:**
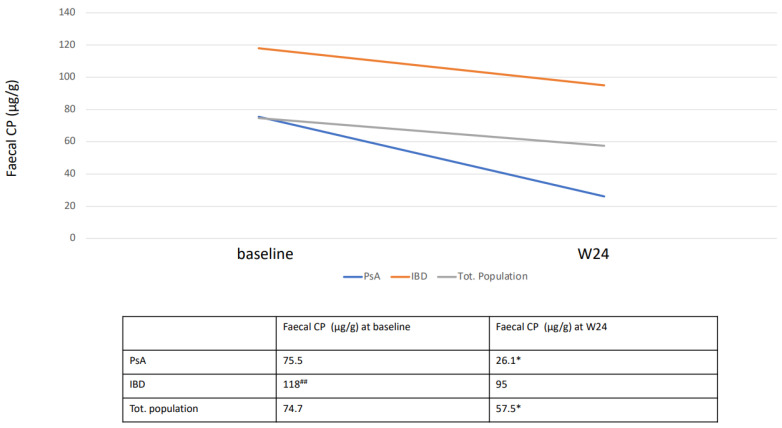
Mean baseline and W24 faecal CP levels stratified by co-morbidities. Data are displayed as mean. Statistical analysis was performed with Wilcoxon matched pairs test: * *p* < 0.05 with respect to baseline; Mann–Whitney test: ## *p* < 0.01 with respect to subjects with PsA.

According to co-morbidities, baseline faecal CP levels were 118 μg/g in patients affected by IBD and 75.7 μg/g in patients affected by PsA. Faecal CP levels of IBD subjects were significantly higher respect to PsA (*p* < 0.01). At week 24, faecal CP levels were reduced in both conditions, but significantly only in PsA subjects (*p* < 0.05) (Figure 2).

**Figure 3:**
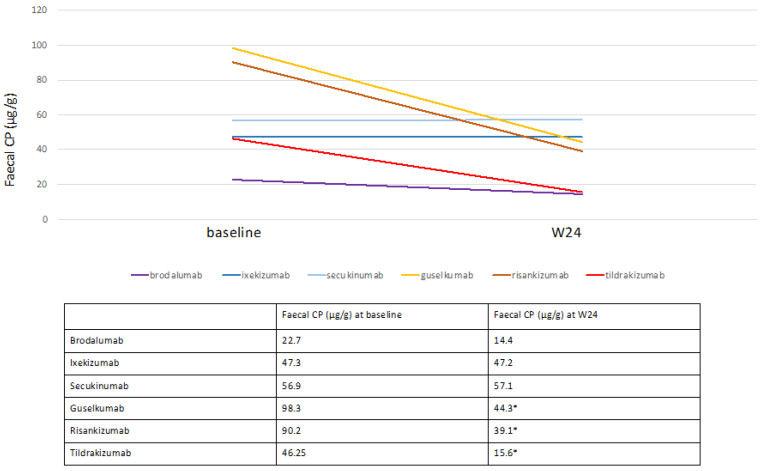
Mean baseline and W24 faecal CP levels stratified by single biological drug. Data are displayed as mean. Statistical analysis was performed with Wilcoxon matched pairs test, * *p* < 0.05.

An analysis of faecal CP values stratified by therapy type was performed. No significant reduction was assessed at W24 for any of the anti-IL17 drugs, whereas a significant reduction was detected for all anti-IL23 drugs (*p* < 0.05) (Figure 3). At W24, a reduction in faecal CP values was also observed for patients on anti-TNF-α, but no statistical analysis was performed for the low number of subjects (data not shown).

In 2 of the 129 patients undergoing therapy with secukinumab, a notable increase in faecal CP at W24 compared to baseline was detected. Afterward, consultations with gastroenterologists and colonoscopy with histological examination of the intestinal samples taken for diagnosis of Crohn’s disease (CD) and ulcerative proctitis, respectively, was performed (Table 2).

## 4. Discussion

In this study, we showed that faecal CP levels in psoriatic patients were higher than the normal range and these levels decreased after 24 weeks of biological therapy, in particular anti-IL23. These results were partially in line with studies from the literature, in fact the increase in CP levels (serum and faecal) were investigated in various inflammatory diseases such as axial spondyloarthritis (axSpA), rheumatoid arthritis (RA), IBD, PsA and psoriasis [30,31,32,33].

Since CP is secreted locally at the sites of inflammation in response to cellular stress or tissue damage, the assessment of serum and/or faecal levels of this protein show important advantages over conventional biomarkers. It is well established that under various clinical conditions, CP levels represent a significant superiority as a biomarker over conventional clinical or laboratory parameters [27].

Jarlborg M. et al. reported that serum CP levels were higher in a series of 1729 patients affected by axSpA, RA and PsA with respect to healthy controls. Moreover, they reported an association between serum CP levels and activity as well as severity of the three clinical conditions [34]. Similarly, a recent study reported an increase in serum CP levels in psoriatic patients compared to healthy controls. The association between serum CP levels and disease severity with reduction after one month of anti-TNFα therapy in psoriatic patients has also been evaluated [35]. As reported by Qian M. et al., the possible reason for this might be that CP is secreted by monocytes and neutrophils, and its concentration reflects the inflammatory status of psoriasis. Since an increase in CP levels is linked to inflammation, its reduction could reflect a sharp decrease in inflammatory cytokines with a consequent amelioration of disease severity [35].

A recent study evaluated the association between faecal CP levels and disease activity in subjects affected by axial SpA. Subclinical intestinal inflammation (assessed by the faecal CP dosage) was found to be more correlated with peripheral joint inflammation than to the axial one in subjects affected by axial SpA [28]. In another study, elevated levels of faecal CP have been described as potentially identifier of patients who are more likely to have SpA already in the unclassified stage of the disease [29]. The link between intestinal and joint inflammation has also been explored in PsA subjects. Adarsh MB et al. reported that faecal CP levels were higher in a series of 50 patients affected by PsA respect to controls. In particular, faecal calprotectin levels were significantly higher in those with a larger body surface area affected and higher PASI score [36]. Other rheumatologic diseases have also been associated to the increase in faecal CP levels, as Sjögren’s syndrome [37].

The utility of faecal CP has been assessed for the diagnosis and monitoring of therapy in patients with IBD [38,39,40,41]. One of the first studies on the utility of faecal CP in the diagnosis of IBD was published in 1997 by Roseth who studied 62 patients with ulcerative colitis reporting higher faecal CP levels than healthy controls [42]. Limburg et al. in a study involving 110 subjects showed that increased faecal CP levels were significantly associated with the presence of colorectal inflammation and that calprotectin was a more sensitive biomarker, especially regarding specificity, for colorectal inflammation at all levels compared to other faecal tests [43]. In a study involving 40 patients with Crohn’s disease, a sensitivity of 85% and a specificity of 81% for faecal calprotectin was reported in the diagnosis of the disease [38]. Similar results were also found in the paediatric population [44,45], and recently, it has been reported that faecal CP can be used to select treatment strategies [32].

Regarding therapy monitoring, Ollech et al. reported the use of faecal calprotectin as a useful marker for the management of patients with Crohn’s disease on ustekinumab therapy [46]. Another study reported the usefulness of the evaluation of faecal calprotectin in monitoring infliximab therapy in patients with IBD [47].

Our findings showed that psoriatic subjects with concomitant IBD had significant higher faecal CP levels respect to PsA ones with reduction by biological treatment even if not significant. In contrast, a significant reduction was assessed in PsA subjects after 24 weeks of treatment. In order to explore if the treatment was able to influence the reduction of faecal CP levels, we stratified patients according to the therapeutic class (anti-IL17 and anti-IL23). Faecal CP values at baseline were higher in patients starting biological therapy with anti-IL23 than in those with anti-IL17 and these values at W24 were significantly reduced in the first group but not into the second. Moreover, we had 2 patients (out of 129) on anti-IL17A (secukinumab) resulting in IBD diagnosis after increase in faecal CP at W24, counselling with gastroenterologists and colonoscopy.

The role of IL-17 in the pathogenesis of IBD is now recognized [27]. It is a cytokine produced by Th17 cells responsible for regulating both adaptive and innate immune defences. There are six types of IL17 (IL17A to IL17F) and five types of receptors (IL17-RA to IL17-RE) [48,49,50]. IL17A has effects on various cells including lymphocytes, macrophages, neutrophils, keratinocytes, fibroblasts, keratinocytes, endothelial cells, epithelial cells and dendritic cells [51]. In the skin, IL17 stimulates the release of pro-inflammatory mediators from keratinocytes with subsequent influx of neutrophils, lymphoid cells, dendritic cells and additional Th17 cells [52].

Th17 cells are prominent in the intestinal mucosal surfaces, where they help maintain intestinal homeostasis and protect against microorganisms [53]. For this reason, suppression of IL17 may interfere with its protective function of the gut, causing onset of infections and explaining the clinical failure of anti-IL17 drugs in the treatment of Crohn’s disease [54]. Several studies have reported IBD exacerbations in association with anti-IL17 drugs for psoriasis. A randomized, double-blind, placebo-controlled Phase II study evaluated the efficacy and safety of secukinumab in patients with Crohn’s disease. One third of patients (31%) discontinued the study prematurely and a planned preliminary analysis showed that the mean reduction in Crohn’s disease activity index (CDAI) score at week 6 was greater in patients treated with placebo compared with secukinumab. Adverse events occurred more frequently in the secukinumab group than in the placebo group, leading to early termination of the study [55]. In an another randomized, double-blind, placebo-controlled phase II study, 130 patients with Crohn’s disease were randomized to receive brodalumab or placebo at baseline and at W4. Remission rates were higher in patients treated with brodalumab compared to the placebo group but a greater CDAI at W6 occurred in the brodalumab group compared to the placebo group. The treatment with brodalumab resulted in a disproportionate number of cases of worsening Crohn’s disease in patients with active disease and no evidence of meaningful efficacy [56].

Cases of onset or exacerbation of IBD have also been reported in patients undergoing therapy with anti-IL17 for dermatological or rheumatological diseases. A systematic revision of thirty-eight randomized studies analysed 16,690 patients treated with anti-IL-17 agents. Twelve cases of new-onset of IBD have been reported in patients receiving anti-IL17 therapy, whereas no cases were reported in the placebo group [50]. In another study, one case of Crohn’s disease was reported with brodalumab out of 1576 subjects, 4 Crohn’s disease and 7 ulcerative colitis with ixekizumab out of 3736 subjects affected by moderate to severe psoriasis. Ixekizumab every 2 weeks resulted in a moderate exacerbation of ulcerative colitis in one patient and a new onset of Crohn’s disease in one patient [57]. At the same time, an analysis of 21 clinical trials, including 7355 patients, examining the incidence of IBD in patients treated with secukinumab suggested a low overall rate of IBD events in these patients [58]. Similar conclusions, regarding the incidence rate of IBD, were achieved in studies MEASURES 1, 2 and 3, phase III studies evaluating the efficacy and safety of secukinumab in ankylosing spondylitis patients [58,59,60,61,62,63,64]. For all the reasons reported above, patients with IBD are contraindicated for treatment with anti-IL17 drugs.

Our evidence that biological therapy was able to reduce faecal CP reflects the fact that the treatment was able to diminish inflammation at the intestinal level, in the whole population as well as in patients affected by PsA. In particular, IL-23 inhibitors were able to induce such a reduction whereas anti-IL-17 agents were unable. These findings suggest the use of faecal CP levels as a valuable tool to explore intestinal inflammation in the management of psoriatic patients under treatment with biologic drugs. Nevertheless, our descriptive study was performed on a relatively small sample size of patients and further investigations will be necessary to validate faecal CP levels to monitor biologic treatment.

## Figures and Tables

**Table 1:** Demographic and baseline characteristics of the study population.

Characteristics	All Patients (n = 129) N, %, Mean ± SD
*Demographics*	
Male	73
Female	56
Age (years)	54 ± 13.6
Weight (kg)	79.5 ± 15.5
Height (cm)	173.8 ± 11.5
BMI (kg/m²)	28.3 ± 5.8
*Co-morbidities*	
PsA	4.65 %
Hypertension	5.42 %
Diabetes	3.87 %
Dyslipidaemia	5.42 %
Inflammatory Bowel Disease	5.28 %
Other	10.8 %
*Psoriasis*	
Family History of PsO	6.20%
PASI	17 ± 4.8
*Previous therapy*	
Bio-naïve	32%
Bio-experienced	68%
≥2 Biological agent	40%

BMI: body mass index, PASI Psoriasis Area Severity Index, PsA psoriatic arthritis, SD standard deviation.

**Table 2:** Patients positive IBD diagnosis after increased faecal CP at W24, consultations with gastroenterologists and performance of colonoscopy.

Pz	Current Therapy	Faecal CP (μg/g) at Baseline	Faecal CP (μg/g) at W24	Diagnosis
1	Secukinumab	49	154	Crohn’s disease
2	Secukinumab	50	110	Ulcerative proctitis

## Data Availability

The data presented in this study are available on request from the corresponding author.
